# product news

**DOI:** 10.1155/S1463924682000509

**Published:** 1982


Journal of Automatic Chemistry, Volume 4, Number 4 (October–December 1982), pages 197–210

### Product news

### Additional reporting system for the H6000

A new and additional results reporting system has been installed in the Haematology Laboratory at Law Hospital, Lanarkshire, UK for use with their Technicon H6000. The report format of results, units and texts is flexible and may be specified by the customer. All data is enclosed within the label dimensions, a maximum of 2 in × 4 in—which is a width of 20 and a height of 24 characters. Results from the Technicon analytical system are printed on self-adhesive peel-off labels which are then attached to the patient's record card. In addition, a master copy of all results is provided on the label-backing paper. The full range of system-warning texts is automatically displayed in the margins between reports when appropriate (LR, LPX, CAL etc.), and is not included on the peel-off label. Upon completion of a sample run, a termination report is printed which summarizes the previous samples which have been flagged for system alarms.

The system uses a compact microprocessor module which includes a buffer memory enabling the storage of up to 15 patients' reports which may be recalled at the operator's request, allowing ample time for paper-roll changes. A printer mounted on a portable base, which may be remote from the parent Technicon system, is included.

*Further information from Technicon Instruments Company Ltd, Evans House, Hamilton Close, Basingstoke, Hampshire RG21 2YE, UK. Tel.: 0256 29181.*

**Circle No. 5 on Reader Enquiry Card**

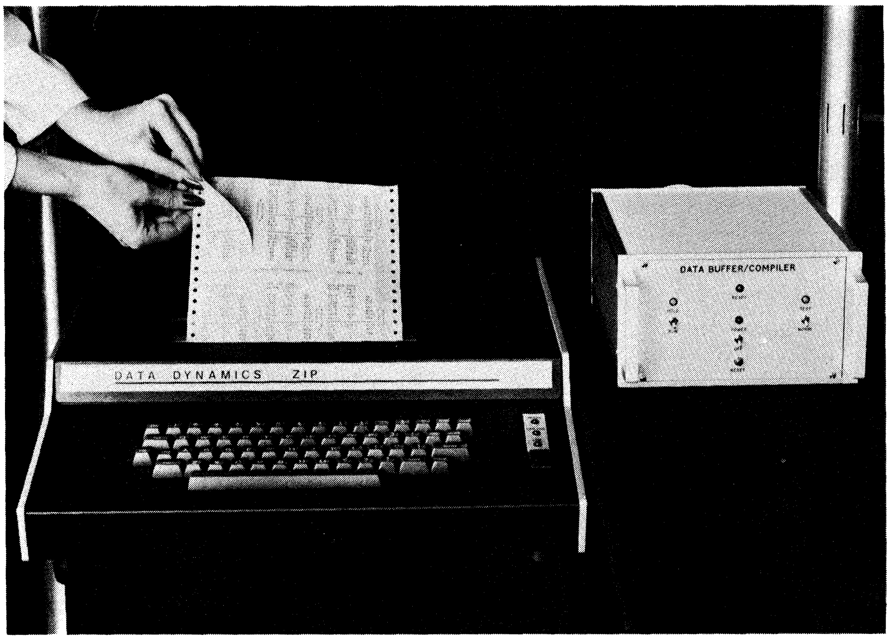

*The new results reporting system for the Technicon H6000 haematology analyser in operation at Law Hospital, Lanarkshire, UK. (Technicon Instruments Company Ltd, Basingstoke, UK.)*

### Automatic controller for chromium rinses

An automatic controller for monitoring and controlling the strength of chromium-containing rinses in metal pre-treatment lines has been announced by Pyrene Chemical Services. The Autobond CR3 controller was developed by the company and is designed to be used with trivalent chromium rinses in spray and immersion pre-treatment plants. Used in conjunction with Pyrene's chemical dosing pumps, the controller ensures optimum performance of the rinse and avoids chemical wastage.

An important market for the Autobond CR3 is the car industry, where a recent change to cathodic electropainting has necessitated the introduction of a chromium-containing rinse at the end of the pre-treatment line, immediately prior to a deionized water rinse. A chromium-containing rinse has proved beneficial to the phosphate-conversion coating process as an additional aid to paint adhesion and increased corrosion protection. Trivalent chromium-based solutions are difficult to control by conventional low-cost conductivity or ion-selective electrode methods, the CR3 overcomes this problem by monitoring the colour of the chromium solution to maintain chemical strength. A robust probe, incorporating a light source and light sensors in two directly opposing arms, is immersed in the solution and the signals generated by the sensors are transmitted to the controller. The degree to which the light is obscured by optical density of the solution is converted to ppm of chromium, or the equivalent titration points, and displayed on a digital read-out by the controller, which also automatically switches the rinse chemical dosing pump on and off as required. The CR3 has an indicated accuracy of $\pm 5\%$ and automatically corrects the reading to allow for build-up of contaminants in the solution. This measurement method is absolute and there is no need for periodic titration to check the digital read-out.

Mounted alongside the digital read-out is a 10-bar illuminated display, which indicates a gradual build-up of contaminants. When eight or more lights are on, a red light warns that the solution needs to be changed, as it has become too dirty for efficient rinsing. The controller also includes circuitry for automatically overflowing the tank to maintain the level of contamination within acceptable limits. In addition to controlling solution strength, it is usually important to maintain a pH of between 4 and 5. If the tank is continually made up with tap-water, for example, the alkalinity of the rinse may rise, but this can be compensated for by adding a compatible acid. For this purpose, the CR3 is fitted with pH monitoring and control of a separate acid-dosing pump.

*Further details can be obtained from Pyrene Chemical Services Ltd, Ridgeway, Iver, Buckinghamshire SL0 9JJ, UK. Tel.: 0753 651812.*

**Circle No. 6 on Reader Enquiry Card**

### Ion analysis

The Opti-Ion series of detectors (Dionex) is intended for work on transition metals, UV/vis absorbing and some non-absorbing compounds and amino-acids. One of the instruments, the Opti-Ion/UV-vis detector, can be used with ion chromatographs and with liquid chromatographs. When combined with a Dionex Series 2000i Ion Chromatograph, it offers significant advantages in the chromatographic capabilities to separate and determine transition metals and compounds. The detector employs a single deuterium source and a series of easily selected filters to provide capability in the 200–750 nm region. So the analyst is able to choose the proper wavelength for most applications without having to buy an expensive monochromator and without having to change filter cartridges. The Opti-Ion/Fluor detector, which uses fluorescence principles, is designed for use with a post-column reactor to provide high-sensitivity detection of amino-acids and polyamines.

*More information from Dionex (UK) Ltd, First Floor, The Parade, Frimley, Surrey GU16 5H7, UK. Tel: 0276 29771. Or from the Dionex Corporation, 1228 Titan Way, Sunnyvale, California 94086, USA.*

**Circle No. 7 on Reader Enquiry Card**

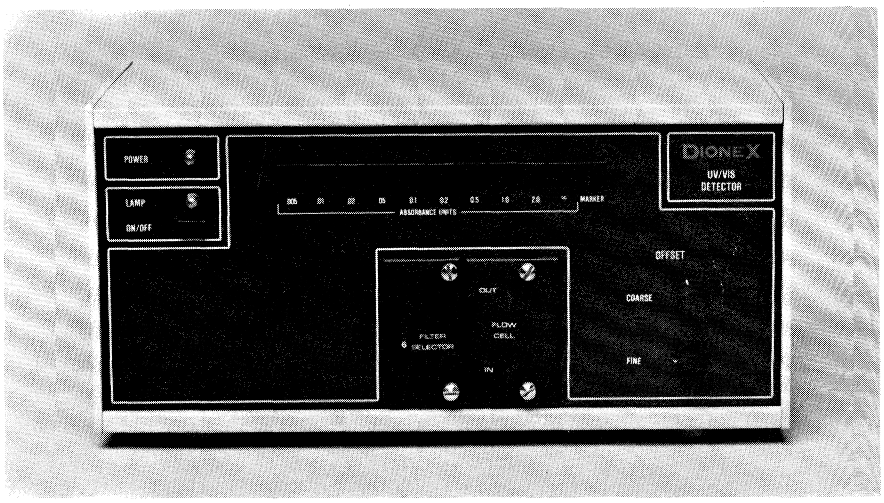

*The Opti-Ion/UV-vis detector, a part of the Opti-Ion series, which when combined with Dionex's ion chromatographs and column technologies provide total ion analysis capabilities. (Dionex, Frimley, UK and Sunnyvale, USA.)*

### Coagulation tests on the ACA

Du Pont have added three fully automated coagulation tests to their ACA (Automatic Clinical Analyser): for antithrombin III (AT III), fibrinogen and plasminogen.

Serious haemostatic problems have traditionally been evaluated with the standard laboratory tests of bleeding time, platelet count, prothrombin time (PT) and activated partial thromboplastin time (APTT). While these tests help define the general dimensions of a patient's problem, the more specific AT III, fibrinogen and plasminogen assays assist in pinpointing causes and provide a more accurate picture of haemostasis. Automation of these tests means that results can be available on a STAT basis. Also, ACA results are consistently precise and accurate, unlike manual methods, which can produce more variable results.

The AT III assay provides insight into the status of an important blood component that protects against thrombosis, and the test is recommended where a patient has a high thromboembolic risk: women taking oral contraceptives and patients over 40 about to undergo surgery for example. An AT III assay also helps to confirm a diagnosis of disseminated intravascular coagulation (DIC) and to monitor the effect of heparin therapy.

The immediate availability of fibrinogen measurements on the ACA makes the fibrinogen test valuable as a supplement to determine PT and APTT; it can also be used as a monitoring tool. The fibrinogen test aids in the diagnosis of DIC in patients who have conditions carrying an increased risk, such as cancer, obstetrical complications, extensive trauma and prolonged shock.

The ACA test for plasminogen, together with AT III, may be useful in diagnosing suspected DIC when the fibrinogen level is normal. And plasminogen testing is useful in diagnosing unexplained recurrent thrombophlebitis and is valuable in monitoring the effectiveness of streptokinase therapy. The test may also complete the clinical picture of a patient with a tendency to bleed or clot.

Du Pont's ACA is now capable of performing 45 different tests; more coagulation tests are being developed for the analyser.

*Details from Du Pont de Nemours International S.A., PO Box, CH 1211 Geneva 24, Switzerland. Tel.: 022 378111.*

**Circle No. 8 on Reader Enquiry Card**

### Metal determination

Routine parts per billion detection of such metals as iron ($Fe^{2+}$ and $Fe^{3+}$), nickel, copper, lead and cobalt can be performed with a simple post-column reactor which features a pneumatic pump, mixing tee and a packed-bed reactor in a self-contained unit. The unit is easily installed in a Dionex Series 2000i ion chromatograph which, when combined with the company's 'Opti-Ion' detectors, results in a completely non-corrosive chromatographic system for optimum separation and detection of metals and amino-acids.

*Details from Dionex (UK) Ltd, First Floor, The Parade, Frimley, Camberley, Surrey GU16 5HY, UK. Tel.: 0276 29771. Or from the Dionex Corporation, 1228Titan Way, Sunnyvale, California 94086, USA.*

**Circle No. 9 on Reader Enquiry Card**

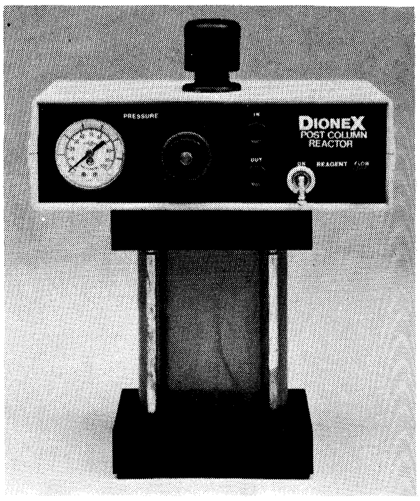

*Dionex's new post-column reactor for determination of ppb of metals.*

### New viscometer for hazardous areas

The Nametre Company's Model 7.006C4P or 7.010C4P direct read-out recording viscometer is now available for high temperature, hazardous area application. The 316 stainless-steel transducer system is contained in a watertight and oiltight 1·8 in thick 316 polished stainless-steel enclosure, which can be as far as 350 ft away from the control room. MTL-161 shunt diode safety barriers provide an intrinsically safe system. The viscosity range is from 0 to 200 000 cps and is selectable by push-button in six linear bands. Digital readout, pipeline or reactor operation, noise filter, 0·2 V d.c. recorder and 4–20 mA control outputs for computer interfacing and density compensation are standard features. Transducers mounted on standard industrial flanges are optional extras. The viscometer has no moving parts which could wear. A unique low-amplitude vibration principle continuously measures the viscous load with a speed of 750 times/s; and measurements are not affected by the flow of material.

*Further information from the Nametre Company, 1778 State Highway 27, Edison, New Jersey 08817, USA.*

**Circle No. 10 on Reader Enquiry Card**

### Automatic centrifuge

An advanced version of Beckmann-RIIC's J2–21 centrifuge, the Model J2–21M, has just been put on the market. The new features of the J2–21M include microprocessor control, which automates centrifugation and offers a higher degree of flexibility. The system can memorize 10 programs, so that runs can be repeated exactly. A choice of nine acceleration and 10 deceleration rates is offered and automatic temperature compensation can be made after keyboard entry of the name of the rotor to be used. Operating parameters and actual conditions are produced on a clear digital display. The machine has 'Ultra-smooth' drive, which means there are no brushes to wear out or replace; and a frequency-controlled induction motor provides the high torque and power to accelerate rotors smoothly and rapidly . Other advantages of the J2–21M are diagnostic signals, automatic partial vacuum, sturdy components and such safety features as rotor imbalance detection, over-temperature protection and an electronic lid lock. The J2–21M accepts 11 rotors (like the J2–21) ranging from versatile fixed angle and swinging bucket rotors to the special elutriator rotor designed for harvesting living cells. The centrifuge's maximum speed is 21 000 rpm and its maximum force is 51 500 g.

*Details from Beckman-RIIC Ltd, Progress Road, Sands Industrial Estate, High Wycombe, Buckinghamshire, UK. Tel.: 0494 41181.*

**Circle No. 11 on Reader Enquiry Card**

### Single/dual data processor for chromatography

The C-R2A computing integrator from Shimadzu offers single- or dual-channel operation with BASIC programming (with 4k RAM in the C-R2A and 20k in the C-R2AX). It can handle up to 1900 peaks with the full range of chromatographic calculations, including multipoint calibration. Special features include reprocessing the chromatogram and recalculation of the results; and 'Alpha Graphic' display of either, or both, chromatograms can be presented as they emerge on the printer plotter and VDU. As an alternative to printing of retention time, peaks can be identified by name with a time scale along the bottom of the chromatogram. The C-R2A will link to computers through an RS232 interface and to the larger Shimadzu GCs and LCs for total operational control.

Finally, through integral NiCad cells, the C-R2A has 2 months' memory protection against loss of mains power. The manufacturer offers a comprehensive range of accessories, including tape and disc storage, current loop interfaces, and GPC modification.

*Further information from the UK distributor: Dr Norman Dyson, Dyson Instruments Ltd, Sunderland House, Station Road, Houghton-le-Spring, Tyne and Wear DH5 0AT. Tel.: 0783 260452.*

**Circle No. 12 on Reader Enquiry Card**

### 'Air-Stream' temperature controller

A directed air stream of 1 SCFM controllable over the range of −85°C to +100°C is being sold by FTS Systems Inc. The air-stream technique provides a way of controlling samples' temperature without interfering with visual observation or physical measurements. The technique is particularly successful when low temperatures are required: the air is dry and will prevent condensation on the specimen. The new unit is described as particularly useful for routine crystal structure analysis since it can be used for specimen temperature control for investigations in X-ray diffraction, NMR, ESR, optical microscopy and infra-red diffraction. The Air-Stream system has a mechanically refrigerated delivery line which permits air delivery to remote locations up to 10 ft without loss of efficiency. A digital indicator controller provides continuous monitoring and temperature control to ±0·1°C.

*Further information from FTS Systems Inc., P.O. Box 158, Route 209, Stone Ridge, New York 12484, USA.*

**Circle No. 13 on Reader Enquiry Card**

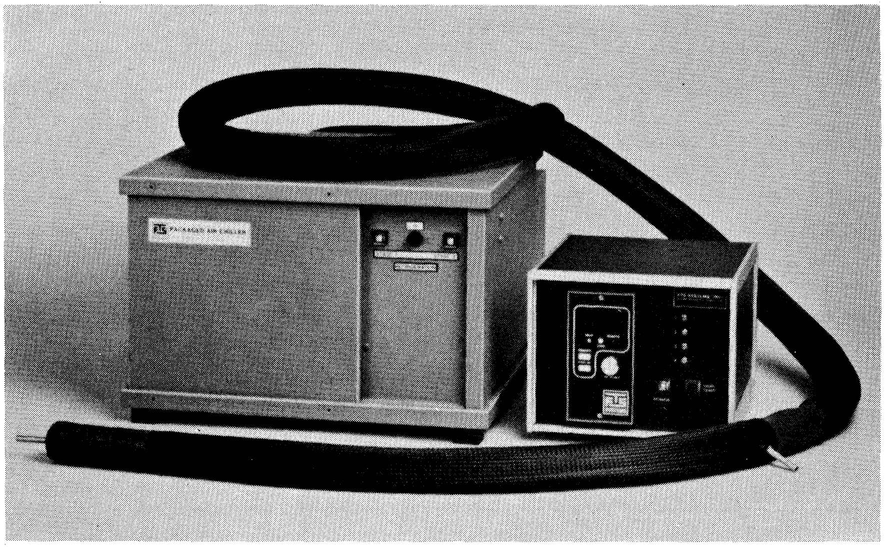

*The Air-Stream temperature controller for crystal structure analysis. The unit controls over the range −85°C to +100°C. (FTS Systems Inc., Stone Ridge, USA.)*

### Flat-surface pH electrode

The Fisher 'Flat-Surface Polymer Combination Electrode', which can be used to measure pH on most moist flat surfaces, is described in a new bulletin (No. 600). The electrode can be used for liquid film, soil samples, meat, fruit, cheese, bacterial plates and in liquid samples, even in liquid beads as small as 10 $\mu$l. And it takes less than 30 s to provide a reading once its flat-surface sensing membrane is pressed to a sample surface. The electrode covers the full 0 to 14 pH range, with repeatability better than $\pm 0{\cdot}05$ pH, on samples from $+10°$ to $+80°$C. The electrode features a rugged polymer body, which is chemically resistant; a porous ceramic plug that serves as a liquid junction; and a 76 cm long lead, terminated in standard American connectors.

*For the bulletin describing the electrode write to Fisher Scientific Company, 711 Forbes Avenue, Pittsburgh, Pennsylvania 15219, USA.*

**Circle No. 14 on Reader Enquiry Card**

### Video + infra-red spectroscopy

Pye Unicam's SP3-080 is an infra-red spectroscopy data terminal used with the company's SP3 series of ratio-recording instruments. The SP3-080 can now be fitted with a video printer; this facility enables the contents of any display on the VDU to be permanently recorded on the printer. A video printer can be selected to give a format size ideal for attaching to an analytical report. Suitable printers available include the Axiom 850 and the larger (A4) Axiom EX 1650, also the more sophisticated Versatec video printer designed for multi-instrument applications, which accepts up to eight video inputs.

*Details from Pye Unicam Ltd, York Street, Cambridge CB1 2PX, UK. Tel.: 0223 358866.*

**Circle No. 15 on Reader Enquiry Card**

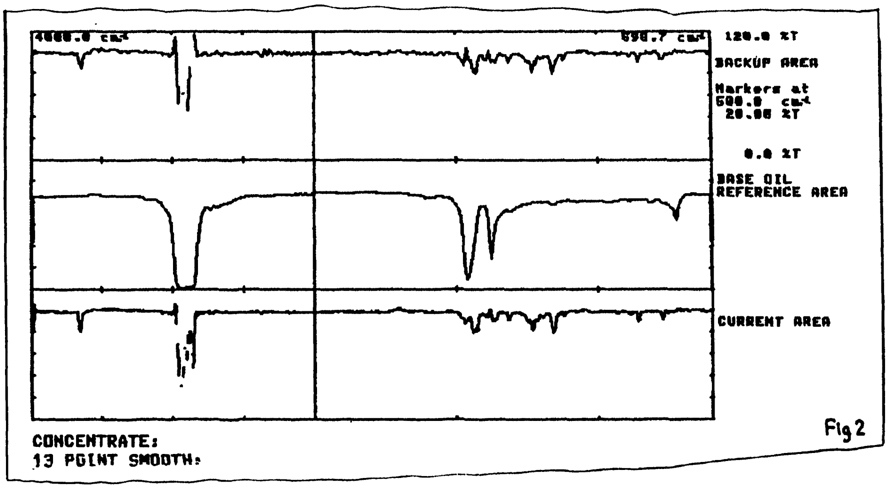

*The SP3-080's infra-red video-printer facility: triple spectrum display after digital smoothing. (Pye Unicam Ltd, Cambridge, UK.)*

### Diaphragm pump for corrosive liquids

The Pump and General Engineering Company recently announced a stainless steel, 2 in diaphragm pump for any industry involved in the moving viscous, corrosive liquids. The diaphragm pump is light and its competitive price has been achieved by tooling for volume production of the casing, and other parts in contact with the liquid, as stainless-steel pressings; other components are cast in anticorodal aluminium. The standard material for the diaphragm and valves in Neoprena, but a number of options are available to suit the material being pumped. Rapid self-priming (from $4{\cdot}5$ m, in around 30 s), minimal maintenance by unskilled labour, and the ability to operate dry for unlimited periods are among the advantages claimed for the pump, which will deal with solid bodies up to 25 mm in diameter. Its weight, without motor, is $12{\cdot}2$ kg.

*Details from Pump and General Engineering Company Ltd, The Ride, Ifold, Billingshurst, West Sussex RH14 0TF, UK. Tel.: 0403 752889.*

**Circle No. 16 on Reader Enquiry Card**

### Anaemia control

'Lyphochek Anaemia Control' is a human-serum based control material designed for haematology laboratories. The serum contains clinically significant levels of vitamin B-12, folic acid, ferritin and transferrin; serum iron, UIBC, TIBC and percentage saturation measurements are also present. The new product is described as making a unique contribution to the control of assays in the diagnosis of anaemic states and, when used in conjunction with Bio-Rad's Lyphochek Radioimmunoassay Controls, it provides total quality-control. The product is freeze-dried and manufactured to rigid quality-control standards; vial to vial stability and consistency of the product are guaranteed by Bio-Rad.

*More information from Bio-Rad Laboratories Ltd, Caxton Way, Holywell Industrial Estate, Watford, Hertfordshire WD1 8RP, UK. Tel.: 0923 45517.*

**Circle No. 17 on Reader Enquiry Card**

### Extending gas chromatograph capability

A circuit board has been developed by Pye Unicam for switching the input of an integrator to a number of chromatograph outputs. Many chromatography applications benefit from the use of an outlet splitter after the column; this allows the measurement of selected components of the sample on different detectors in order to obtain the best possible analysis, for example flame-ionization/thermal conductivity, flame-photometric/thermal conductivity, flame-ionization/electron-capture etc. It is clearly uneconomic to use a separate computing integrator for each detector. So Pye Unicam have developed a circuit board which can be fitted to their Series 304 and PU 4500 gas chromatographs giving automatic switching of an integrator from one detector to another, controlled by the timed event outputs of the integrator. The detectors can actually be switched several times, the number of switches being limited by the number of timed events available from the integrator.

*A brochure called 'Automatic Gas Chromatography—Applications', which describes a number of chromatography examples using these output switching techniques, is available from the manufacturer: Pye Unicam Ltd, York Street, Cambridge CB1 2PX, UK. Tel.: 0223 358866.*

**Circle No. 18 on Reader Enquiry Card**

### Four-output high voltage power-supply system

The 'Quadrupower' or Bertan LS-10 is designed for applications requiring master-slave or independent multi-output operation; it is suitable for powering quadrupole optics or the pre- and post-accelerator sections in ion-implantation equipment. The LS-10 provides two 0 to +10kV at 2·5 mA and two 0 to −10kV at 2·5 mA supplies. Front-panel switches allow selection of master-slave tracking or independent control operations. Regulation of all outputs is less than 100 mV for $\pm 10\%$ line change and 500 mV for NL–FL or FL–NL variations; ripple is less than 2·5 V pk-pk. Independent or tracking mode operation can be remote resistance or voltage programmed and remote monitoring is included for all four outputs. The outputs are short-circuit and over-voltage protected. An IEEE-4888 interface will be available shortly. The LS-10 is fully solid state and features plug-in printed circuit boards and encapsulated high-voltage circuitry. Input power is 115/230 V, 50/60 Hz.

The instrument is available from stock but can be customized: alternative controls, programming, output voltages and interfaces are possible.

*Details from Howard Rachlin, Bertan Associates Inc., 3 Aerial Way, Syosset, New York 11791, USA.*

**Circle No. 19 on Reader Enquiry Card**

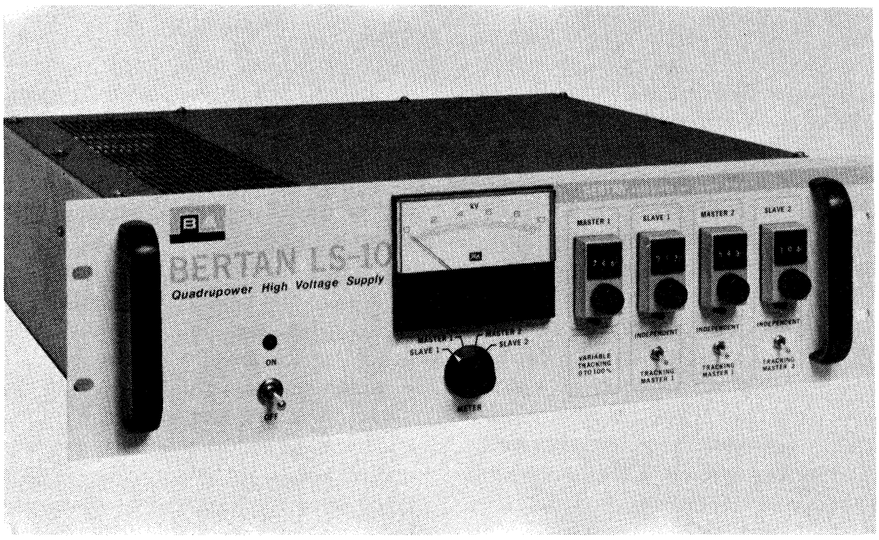

*The Quadrupower—a four output, high voltage power-supply system. (Bertan Associates Inc., Syosset, USA.)*

### High-sensitivity analysis for phenols in water

A complete HPLC system for the analysis of priority phenols in water (the concentration of phenols as by-products and degradation species in waste-water is now regulated) has been announced by Anachem. HPLC with electrochemical detection offers many advantages over colorimetric detection and other methods. Phenols are easily oxidized at a glassy carbon working electrode and thus may be detected to low picogram levels in aqueous samples. The isocratic HPLC system recommended for this analysis includes the Model 302 pump, injection valve, column and electrochemical detector. Optional data handling and automatic sample-injection are available where large numbers of samples are generated. A further option allows 'on-line' trace enrichment of very low concentration samples prior to LC analysis, this technique being particularly applicable to tap-water analysis. Complete application notes are available covering the HPLC/ECD analysis of a wide range of phenols, cresols and chlorophenols.

The pumping system included in Anachem's HPLC system is manufactured by Gilson Medical Electronics (72 rue Gambetta, Villier-le-Bel, France) and the detector is made by Bioanalytical Systems Inc. (1205 Kent Avenue, Purdue Research Park, West Lafayette, Indiana 47906, USA).

*For details and demonstration contact Anachem Ltd, 15 Power Court, Luton, Bedfordshire LU1 3JJ, UK. Tel.: 0582 35252.*

**Circle No. 20 on Reader Enquiry Card**

### Fluorescence detector brochure

A six-page brochure, which describes their series of fluorescence detectors for HPLC, is offered by Kratos Analytical Instruments. Included in the brochure are descriptions of a spectrofluorometer (model 970) and a filter fluorometer (model 950). The model 970 includes such features as: continuously variable excitation wavelength (190–700 nm), and an ultra-micro 5 $\mu$l flowcell with high-efficiency emission energy collector. And the model 950 is a low-cost filter fluorometer suited for a variety of applications. Excitation wavelengths from 214 to 545 nm are available.

*A complete list of the detectors' specifications is published; copies from Kratos Analytical Instruments, 24 Booker Street, Westwood, New Jersey 07675, USA.*

**Circle No. 21 on Reader Enquiry Card**

### Temperature-sensing probes

Grant Instruments' range of temperature-sensing probes is now being offered to OEM (original equipment manufacturers) and to end-user customer. Their range of probes use three basic types of sensor, the thermistor, thermocouple and platinum resistance, which together provide a combination of more than 50 types. Two sizes of thermistor are used, both providing accurate temperature sensing over the range −50°C to +150°C. The thermocouples, chromal-alumel, copper-constantan and iron-constantan provide an overall temperature range of −250°C to +1250°C; accuracy is dependent on the sensor tolerance and cold-junction compensation. Grant's p.t. 100 platinum resistance sensor can be used between −150°C and +300°C; it has a slower response than the thermocouples, but is more accurate.

The company have 13 types of probe in current production including probes with stainless-steel sheaths; two catheter probes embedded in flexible nylon tubing of 0·4 mm and 0·8 mm diameter; two hypodermic probes, in needles 0·4 mm and 0·6 mm diameter; one surface probe, using an epoxy-coated copper disc; an air-temperature probe with a removable globe; an unprotected thermocouple, and a mineral-insulated probe with a stainless-steel sheath. The probes are available with co-axial and non-co-axial PVC, nylon sheath and PTFE cables of lengths up to 50 m (up to 500 m can be supplied when thermistor sensors are used).

*Details from Grant Instruments (Cambridge) Ltd, Barrington, Cambridge CB2 5QZ, UK. Tel.: 0763 60811.*

**Circle No. 22 on Reader Enquiry Card**

### Liquid head assembly for HPLC system

DuPont is offering a new head assembly for its Series 8800 HPLC pump module, which can be converted in a few minutes from analytical to preparative operation. DuPont's 8800 Series HPLC system is thus the first to offer single-pump, binary or multi-solvent gradient capability for both analytical and preparative separations. The self-contained head assembly can operate at back-pressures high enough (up to 200 bar) to allow the preparative pump to work with micro-particulate analytical columns, thus minimizing solvent consumption during method development. This high back-pressure capability also permits use of micro-particulate packings for preparative separations in reversed phase chromatography. The use of a 'Zorbax' preparative column provides a large sample capacity in a minimum solvent volume, thus reducing the work required to recover the sample after collection.

With the preparative head installed, the Du Pont pump also provides the high flow rates (up to 40 ml/min) needed for high sample throughput on an analytical time scale using preparative columns. This capability enables maximum advantage to be derived from the use of micro-particulate columns and produces high resolution for difficult separations and sharp peaks for concentrated fractions.

*For further information contact Du Pont (UK) Ltd, Wedgwood Way, Stevenage, Hertfordshire SG1 4QN, UK. Tel.: 0438 734688.*

**Circle No. 23 on Reader Enquiry Card**

### Automatic wet digestion system

The automatic VAO wet digestion instrument can be used for mechanized chemical analysis and trace analysis of organic and biological samples. The digestion parameters are strictly reproducible and both digestion temperature and period can be varied over a wide range: for example from room temperature up to 300°C (a special version can operate up to 400°C); digestion period is selectable between 10 min and 5 h. Sample throughput is continuous. All types of wet digestion can be accomplished with the VAO: nitric acid, sulphuric acid, chloric acid, perchloric acid, hydrogen peroxide and mixtures of these reagents. The manufacturer expects the instrument to be used in the analysis of food stuff, fodder and fertilizers; raw organic, intermediate and final products in chemistry and pharmacy; blood sera, urine and tissues in hospitals and medical laboratories; and waste water, soil samples etc. in environmental laboratories.

*Details on the VAO instrument from Paar Scientific Ltd, 594 Kingston Road, Raynes Park, London SW20. Tel.: 01 542 9474.*

**Circle No. 24 on Reader Enquiry Card**

### Threadless spray nozzle

A high-pressure threadless spray for applications ranging from industrial steam-cleaning and vehicle-washing equipment, through to paint chemicals and crop-spraying systems has been announced by Clayton-Heyes Engineering. Nozzle replacement takes only seconds to perform using a simple hand-tool, whilst high-pressure jets reduce the risk of blockages. The spray has options for solid or hollow-cone jets and is designed to operate at pressures up to 2000 p.s.i. Benefits claimed by the manufacturer include increased capacity stemming from work-pieces being rapidly indexed through industrial spray-plant equipment and, for horticulture and agricultural industries, ground is covered more quickly without loss of area cover efficiency. The spray body and nozzle are made from stainless steel. Each replaceable nozzle is firmly held in place and cannot be dislodged by fluid pressure or removed by hand without using the hand-tool provided.

*Full details from Clayton-Heyes Engineering Company Ltd, Abbeydale Works, Woodseats Road, Sheffield S8 0PF, UK. Tel.: 0742 51004.*

**Circle No. 25 on Reader Enquiry Card**

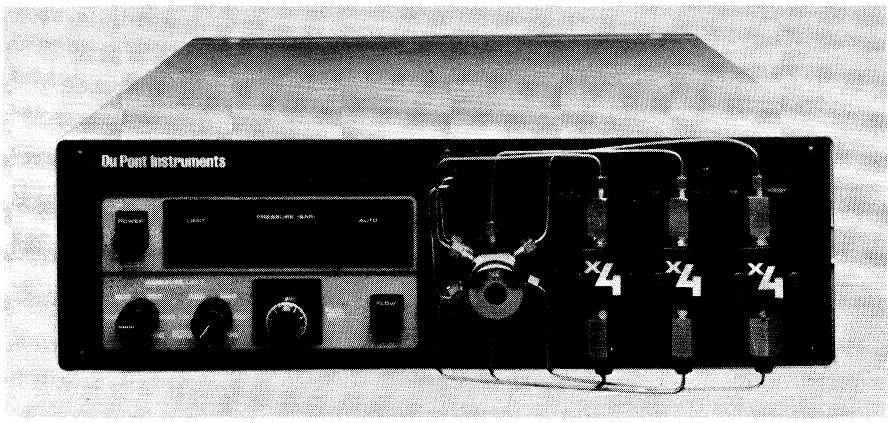

*Du Pont's Series 8800 pump module, showing (right) the new preparative head assemblies. (Du Pont de Nemours International S.A., Geneva.)*

### Brochure about rapid-scanning detector for HPLC

A brochure describing their Model 165 variable wavelength UV/vis detection system for liquid chromatography is available from Beckman-RIIC. The Model 165 includes a microprocessor-controlled monochromator which provides qualitative spectral information over the UV/vis range to assist in the identification and measurement of compounds eluting from the LC column. Dual-channel monitoring of absorbance at any two selected wavelengths—LX 195 and 600 nm—is a feature of the machine. This avoids the risk of not detecting compounds which do not absorb strongly at the more normal detector wavelengths. Time-programmable wavelength changes can be made to maximize detector response. A rapid-scan feature combines a very fast scanning speed with automatic concentration correction and a fast time constant in order to produce useful spectral information on each peak, either automatically or manually. The requirement to stop flow is eliminated. And a ratio channel provides a time-corrected plot of the ratio of absorbances at two pre-selected, calibrated wavelengths. This 'ratiogram' can be of great assistance in confirming peak identity or purity.

*Copies of the brochure from Beckman-RIIC Ltd, Progress Road, Sands Industrial Estate, High Wycombe, Buckinghamshire, UK. Tel.: 0494 41181.*

**Circle No. 26 on Reader Enquiry Card**

### Perkin-Elmer 1957–1982

Perkin-Elmer Ltd celebrates 25 years of manufacturing analytical instruments this year. The company was set up in Beaconsfield as a small-scale operation in 1957; it is now a leading supplier exporting over 80% of its production. The number of employees has increased from the original 10 to almost 700, and the facilities have expanded from an initial 10 000 $ft^2$ to 238 000 $ft^2$, split between Beaconsfield and Llantrisant, South Wales. Highlights of the past 25 years include sales to Russia and China, the Queen's Award to Industry for exports in 1971, and two Design Council awards: one for the F30 gas chromatograph in 1974, the other for the Model 580 infra-red spectrophotometer in 1977.

The first instrument produced at Beaconsfield was the Model 137 'Infracord', an infra-red spectrophotometer; it was followed by a range of infra-red instruments—the popular 57 Series, the 680s and the more recent 983. The company's recent infra-red spectrophotometers, the 780 Series, incorporate the latest technical advances: microprocessor-control, ratio recording and built-in data-processing factilities.

In addition to infra-red machines, Perkin-Elmer Ltd has developed and built a wide range of analytical instruments using ultra-violet, gas chromatography, photoelectron, nuclear magnetic resonance and fluorescence techniques.

*Full details on Perkin-Elmer's new products, below, can be obtained from Perkin-Elmer Ltd, Post Office Lane, Beaconsfield, Buckinghamshire HP9 1QA, UK. Tel.: 04946 6161.*

### Liquid chromatography detectors

The LC-21 conductivity detector and the LC-4B electrochemical detector have recently been launched by Perkin-Elmer. The LC-21 converts any Perkin-Elmer liquid chromatograph into an ion-chromatography system: it detects ions in solution by monitoring changes in electrolytic conductivity. Its wide-range background suppression allows the use of most common buffers without the need for special ion-suppression columns. The LC-21 is available as a detector only or as a system, including a matching ion-exchange column to provide complete anionic separation and detection capability.

The LC-4B provides selective and sensitive analyses of materials which can be readily oxidized or reduced (for example catecholamines, nitrosamines and phenols). The instrument consists of the 'Amperometric Controller' and a thin-layer cell. The new amperometric controller features illuminated status indicators with digital read-out of applied potential, output current and background offset-current. Specially designed protective circuits shield sensitive electronic components from static discharge. Current-to-voltage gain is available from 0·1 to 500 nA/V in 12 steps and a convenient internal electronics test circuit is included. Several units can be combined for multiple-electrode applications.

**Circle No. 27 on Reader Enquiry Card**

### Dynamic mechanical analyser

Perkin-Elmer announce an economical dynamic mechanical analyser module (DMA) designed as an accessory for their TMS-2—a thermomechanical analyser. The DMA can easily be fitted to existing TMS-2 units, or ordered as an integral part of new TMS-2s. The DMA is easy to operate and requires no clamping of the sample; so it can be used to characterize high-modulus thermosets as well as thin films and rubbers. For dynamic mechanical analysis, the fixed force of the TMS-2 is replaced by a magnetically induced force, modulated at frequencies from 0·01 Hz to 10·0 Hz. Force values from 0 to 20 g may be selected. Modulation may be either square wave or sine wave in nature. The DMA module does not inhibit the usual function of the TMS-2, so that thermomechanical and dynamic mechanical measurements may be performed on the same sample sequentially.

**Circle No. 28 on Reader Enquiry Card**

### Thermal analysis software

Perkin-Elmer have added a new package to its range of thermal analysis software. The DTA 1700 Standard provides instrument control, data acquisition, processing, reporting and storage of results from the company's DTA 1700 differential thermal analyser. An analysis library includes routines for peak-area measurement, glass transition evaluation and comparison of thermograms of clays, minerals, cements and glasses.

**Circle No. 29 on Reader Enquiry Card**

### Gas chromatograph

The Open Tubular Column SIGMA 3B is a dedicated gas chromatrograph which Perkin-Elmer are adding to their line of preconfigured routine analysers. The new instrument, fitted with both the 'split/splitless' and 'on-column' injectors, is optimized for open tubular column chromatography. This combination of injection systems provides the analyst with an instrument capable of all open tubular column injection techniques. The firm's newly developed on-column injection system assures leak-free injection. When used with a fused silica column and needle, the new preconfigured SIGMA 3B provides completely inert on-column injection. The Open Tubular Column SIGMA 3B has a year's warranty and is available for immediate delivery.

**Circle No. 30 on Reader Enquiry Card**

### Perkin-Elmer software

PECLS is a data-processing software package which Perkin-Elmer has launched to interface their LS series of luminescence spectrometers with the model 3600 data station. The software allows the analyst to determine instrument parameters, to operate the instrument directly, to program operation to manipulate spectra, to store and retrieve data, and to obtain printed copies of spectra or alphanumeric information using the model 660 printer. PECLS provides the analyst with a powerful tool without the need to learn computer programming. Full instrument control is provided via the data station keyboard, and special-function keys are used to initiate 32 software commands. After selecting the appropriate function key, the analyst simply specifies the operating parameters. Any of the operations actioned by the special-function keys can also be entered by typing the command via the normal keyboard. In addition to these special functions there are seven other commands, which are entered through the keyboard and used for spectral and data manipulation.

A particularly useful feature of PECLS is the ability to string a number of commands together in mini-programs called OBEY files. Such programs allow the analyst to carry out a wide variety of operations with the instrument completely unattended and to provide automatic decision-making based on the results.

**Circle No. 31 on Reader Enquiry Card**

### 3030 AA spectrophotometer

The new Model 3030 atomic absorption spectrophotometer offers video display of analytical data and operating conditions, proven Perkin-Elmer high-performance optics and high-speed computer electronics. The machine has a large 12 in video display unit (VDU) for monitoring an entire AA analysis from start to finish. Analytical information for all elements, peak shape display and calibrations appear on the VDU as required. Optional graphical displays show true analytical signals generated during flame, graphite furnace, cold vapour and hydride-generation techniques. With the operating system and analytical data stored on floppy disc, upgrading is easy. Up to 30 methods can also be stored on each floppy disc. 'Soft' keys for entry of parameters and method selection reduce the number of function keys to a minimum and provide logical and easy instrument set-up. The 3030 uses quartz-coated reflective optics for efficient operation over a wavelength range of 190 to 870 nm with a resolution of 0·07 nm. Two wavelength scan speeds are built-in: 1 and 5 nm/min. A dual-blazed grating provides high energy throughput over the entire wavelength range for low base-line noise. All Perkin-Elmer AA accessories are compatible with the 3030. In addition, it has an optical two-way RS 232 interface to connect the spectrophotometer to an external computer, and a printer is available for hard copy.

**Circle No. 32 on Reader Enquiry Card**

### Automatic analyser for naphtha-type samples

Market research in refineries and other sections of the petrochemical industry has shown a growing need for the replacement of ageing equipment and for greater flexibility and automation in naphtha analysis.

Perkin-Elmer, in co-operation with a Dutch company, Analytical Consumables, have developed a special naphtha analyser based on the SIGMA range of gas chromatographs. The following analysis options are available: PNA—paraffins, naphthenes, aromatics; PINA—paraffins, iso-paraffins, naphthenes, aromatics; PONA—paraffins, olefins, naphthenes, aromatics; PIONA—paraffins, iso-paraffins, olefins, naphthenes, aromatics. Complex mixtures like naphthas cannot be separated on a one-column system. The naphtha analyser contains two separate gas chromatographic systems interconnected via a trap. The system permits quantitative analysis per carbon number of paraffins up to C11, naphthenes up to C10–C11 and aromatic components up to C10. All components with a boiling point above 200°C are summed. All stages of the naphtha analysis are performed by timed-event relays, which can be provided by a SIGMA data station or by the user's own computer system.

**Circle No. 33 on Reader Enquiry Card**

### Electrolytic conductivity detector

The Hall Electrolytic Conductivity Detector is now being sold by Perkin-Elmer for use with the SIGMA 3B gas chromatograph. The Hall detector has four specific modes of operation: halogen, nitrogen, sulphur and nitrosamine. It can be adjusted from one mode to another by appropriate changes of reaction gas, reactor temperature, electrolyte composition, or the scrubber to eliminate specific reaction products. Perkin-Elmer consider the instrument effective for analysing sulphur in hydrocarbon compounds (such as natural gas and pesticides). In the nitrogen mode its applications include analysis of pesticides in soils, vegetables and animal tissue. The halogen mode is designed for sensitive analyses of chlorinated pesticides and polychlorinated biphenyls (together with a purge-and-trap method of sample concentration the detector is ideal for determining trihalomethanes in drinking-water). And in its nitrosamine mode it will detect nitrosamines in the presence of other nitrogen-containing compounds.

**Circle No. 34 on Reader Enquiry Card**

### Long-life thermocouple pyrometers

H. A. Wainwright & Co. Ltd have sent *JAC* details of their range of long-life thermocouples; the devices are described as compact and rugged and suitable for a wide range of industrial and commercial applications. The company offer four subsurface pyrometers which operate over large temperature ranges: −75°C to 871°C, 0–1400°C and 0–2500°F. Some models, such as the Pyro Surface Pyrometer use no batteries and are available with a choice of four extension arms and 16 different interchangeable thermocouples.

H. A. Wainwright can supply 'bare metal' and 'protected' thermocouples, these are completely interchangeable without calibration.

The company's six non-contact pyrometer types are intended mainly for measuring high temperatures. The series includes radiation thermometers for measuring kiln temperatures over 1000°F in forging operations and a variety of optical, micro-optical, and automatic optical pyrometers. Six single-, double- and triple-range optical pyrometers are available for measurements above 1400°F; these have an accuracy of 1–2%. The solid state optical Pyro Photo II measures, indicates and records temperatures above 775°C. And there is a micro-optical unit for high-precision laboratory work and plant applications, which has three wide switch-selectable ranges from 700–3200°C. Special extended ranges can be provided to 10 000°C.

Wainwright have two new units: the Pyro 'Temp-Pal' and the 'Temp-Shooter': the 'Temp-Pal' is an advanced digital read-out thermocouple pyrometer for surface and subsurface temperature measurement. It uses a variety of interchangeable thermocouples, it is hand-held and is attached to the thermocouple by a flexible, metal-sheathed cable. The 'Temp-Pal' will run for a year on one replacable 9V battery and works over two temperature ranges. The user can switch immediately between a range of −100°F to +1600°F and a range of −75°C to +871°C. The temperature sensed by the probe is displayed in clear LCD numerals, with an accuracy of ±1% of the reading. This pyrometer is electronically linearized, with automatic cold-junction compensation and a self-checking feature which allows the user to check the ice-point reference for operational integrity.

The 'Temp-Shooter' is an infra-red non-contact pyrometer, which measures temperatures up to 1000°C with an accuracy of 1·5% of full scale. Unique features include an adjustable focus lens, track peak modes of operation and an extremely stable electro-optical system. The unit is powered by a long-life, rechargeable nickel/cadmium 9V battery.

*Further information from H. A. Wainwright & Co. Ltd, 9–11 Farncombe Street, Farncombe, Godalming, Surrey GU7 3BA, UK. Tel.: 04868 28384.*

**Circle No. 35 on Reader Enquiry Card**

### Temperature-controlled baths

The DP series is Grant Instruments' new range of temperature-controlled baths: they have an LED temperature display and feature a high degree of control accuracy and uniformity over the whole working area of the bath. Particular attention has been paid by the manufacturer to the design of the control unit, eliminating all projections from the top and sides. The main controls, together with the LED display, are mounted unobtrusively on the inset control panel. The temperature setting is by thumbwheel adjustment—avoiding the traditional large setting knob, this is fitted with a locking device to avoid accidential movement. The contol unit case is constructed entirely from metal, providing the strength and resilience needed for continuous use. The stainless-steel heater is controlled by a new solid-state system, which was developed specifically for the series. Liquid circulation within the bath is provided by a high-speed stirrer; the stirrer also incorporates a centrifugal pump for circulating the bath liquid through external apparatus so the bath can be used as a temperature-controlled circulator. Bath temperature is continuously monitored on the LED display; by pressing a switch, the display also monitors the set temperature of the bath, this is also used when the temperature control is being adjusted. Five sizes of bath are available: the DP10, 15, 20, 35 and 50l. When used with a CC25 refrigerated cooler unit, the DP baths have an overall temperature range of 0 to 80°C.

*More information from Grant Instruments (Cambridge) Ltd, Barrington, Cambridge CB2 5QZ, UK. Tel.: 0763 60811.*

**Circle No. 36 on Reader Enquiry Card**

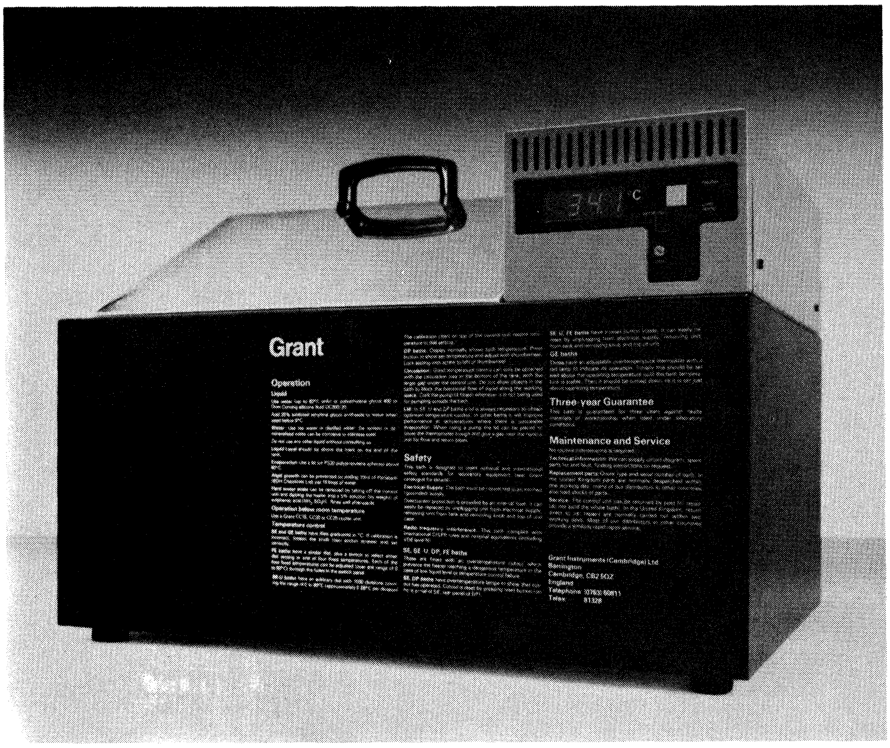

*The DP20—one of Grant Instruments' new DP series of temperature-controlled baths with an LED temperature display.*

### Fluid-flow tubing

The TFE 'Miniature Fluid-Flow Tubing System', made of leak-proof and easy-to-assemble components, allows fluids of all types (gas or liquid/acid or alkali) to be transferred and controlled without loss or contamination. The tubing can be used at high or low temperatures, under pressure, or in a vacuum. The tubing system includes its manufacturer's 'Elast-o-fluor' seal, which permits quick and positive installation of fittings and valves without fumbling for inserts, sleeves, or ferrules. Tubing is simply inserted and the nut tightened. The elastomeric ring in this design maintains a continuous, uniform sealing pressure on the fitting wall, keeping it tight against the tubing, even if the tubing is out-of-round. Components of the new miniature tubing system (tube fittings, plug valves and flexible tubing) are simple in design, rugged and versatile. Fittings include straight unions, reducing unions, elbows, tees, plug valves, male and female luer adapters, crosses, and adapters for threaded pipe; all are made of virgin TFE to give maximum protection from corrosion and contamination. By selecting the appropriate components, a miniature tubing system can be assembled to meet virtually any industrial or laboratory equipment. The system is easy to disassemble. All components can be cleaned thermally or chemically in the most rigorous environments to render them biologically and chemically clean, and they are completely reusable.

*More information from Chemplast, Inc., 150 Dey Road, Wayne, New Jersey 07470, USA. Tel.: 201 696 4700.*

**Circle No. 37 on Reader Enquiry Card**

### Cryogenics for X-ray diffraction

Oxford Instruments has issued a four-page brochure describing their latest cooling systems for X-ray diffraction. Cryostats and coolers are available for reducing the temperature of powdered or bulk crystalline samples and single crystals. The company have 10 years' experience in the field and offer a unique range of cooling systems giving temperatures from 1·5 to 500°K, to suit a wide variety of 2+4 circle diffractometers, goniometers and Weissenberg systems.

*The brochure, 'X-ray Diffraction Systems: Cryogenic Sample Chambers for Cameras and Diffractometers', is available from Oxford Instruments, Osney Mead, Oxford OX2 0DX, UK. Tel.: 0865 41456.*

**Circle No. 38 on Reader Enquiry Card**

### Chemical warehousing and distribution

Total Concept Distribution is a division of the EPS group of companies which is taking contracts for chemical warehousing and distribution. They offer a full service and believe that a number of major chemical manufacturers (they already handle Dow Corning) will be interested in their services because of the costs and effort involved in operating an in-house system. Their service begins at the end of the production line and includes expert storage of hazardous material, order-picking, delivery and stock control.

*Details from Neil Backwith, Countrywide Communications Ltd, 23 West Bar, Banbury, Oxfordshire OX16 9SH, UK.*

**Circle No. 39 on Reader Enquiry Card**

### Silicon photodetector

Kratos's D505-1 silicon photodetector is a UV-enhanced detector with a UV-grade quartz window. The response profile of the D505-1 is flat (± 5%) between 200 and 1080 nm. The D505-1 includes an integral housing with adapters that permit direct attachment to Kratos monochromators.

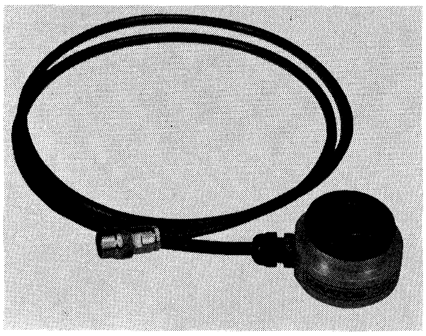

*Kratos Analytical Instruments' D505-1 silicon photodetector.*

The flat response of the detector means it is very suitable for application as the detector element in a spectroradiometer. The D505-1 can also be used in broad-band applications. The photosensitive surface area of the detector is 33 $mm^2$ (5·9 mm x 5·9 mm). Kratos also manufacture germanium detectors.

*Further information from Kratos Analytical Instruments, 170 Williams Drive, Ramsey, New Jersey 07446, USA. Tel.: 201 934 9000.*

**Circle No. 40 on Reader Enquiry Card**

### Ion-exchange resin selection pack

Laboratories performing chromatography, electrophoresis, immunology and quantitative analysis need ion-exchange products for deionization, desalting and other ionic separations. And because the choice of methods available for any of these applications is varied, the cost of purchasing materials to try all the alternatives can be inhibitive to ultimately achieving the best method. Bio-Rad Laboratories, therefore, have introduced an ion-exchange selection pack, which is designed as an introductory range of resins in 100 g quantities. The range includes: 100 g of AG50W-X8 100–200 mesh cation exchange resin; 100 g of AG1-X8 100–200 mesh anion exchange resin; 100 g of AG501-X8 20–50 mesh mixed bed resin for deionization; and 100 g of Chelex 100 100–200 mesh chelating resin.

All the resins are composed of a highly purified styrene divinylbenzene matrix with an appropriate counter-ion. In addition to the resins, the kit contains a 40-page basic manual on ion-exchange theory and practice.

### *Heparin affinity gel for protein purification*

Bio-Rad have also announced an addition—Affi-Gel Heparin—to their range of affinity gels. The new gel is a ready-to-use affinity support for purifying a wide range of proteins. It provides a rapid, one-step purification of coagulation factors and other plasma proteins, polynucleotide polymerase, nuclease, lipase, lipoproteins and proteases. The sample is applied and unbound proteins eluted with low salt buffer; by increasing the salt concentration the bound protein is eluted. A mixture of bound proteins may be selectively eluted using a salt gradient or, alternatively, using selected heparin concentrations. Regeneration of the column is simply achieved by 2·3 bed volumes of 8M urea, 1·5M NaCl in PBS.

*More information about their resins and gels from Bio-Rad Laboratories Ltd, Caxton Way, Holywell Industrial Estate, Watford, Hertfordshire WD1 8RP, UK. Tel.: 0923 40322.*

**Circle No. 41 on Reader Enquiry Card**

### Accessories for chromatography

Additions to the Trilab and Trojan range of chromatography data systems have been announced by Trivector Scientific Ltd. Mini-floppy disc drives, for example, are now available for the Trilab; providing nearly 1 megabyte of backing store. This enables storage of raw and processed data, operating and analysis methods and programs, and BASIC and BASIC programs on disc. A high-speed printer/plotter, capable of being driven by the standard chromatography routines and by BASIC, has also been added to Trilab. The Trojan data system has new chromatography search routines, which enable a data library on disc to be rapidly scanned for chromatogram matching.

### *ERMA degasser*

The ERMA range of in-line degassers for HPLC or other liquid-flow techniques will in future be distributed in the UK by Trivector. The ERMA systems enable multiple-solvent streams to be degassed effectively and economically, without the expense of helium degassing or the danger of vacuum degassing.

*Details from Andrew Blow, Trivector Scientific Ltd, Sunderland Road, Sandy, Bedfordshire SG19 1RB, UK. Tel.: 0767 82222.*

**Circle No. 42 on Reader Enquiry Card**

### Flame photometer

As an addition to their range of flame photometers, Instrumentation Laboratory have just released the new microprocessor-controlled IL943. The IL943 measures the concentration of sodium, potassium and lithium in serum, plasma, urine and other biological fluids. Caesium is used as the internal standard allowing fast, reliable lithium determinations and reduced instrument maintenance. The IL943 incorporates a newly designed self-priming piston dilutor, allowing analysis with as little as 20 μl of sample without the need for a supply of distilled water. Microprocessor control gives fully automated operation using a spill-proof membrane keyboard, and results are shown on four-digit LED displays. The instrument can be supplied with or without a printer and an optional integral automatic sampler allows analysis at a rate of 100 samples/h, but still permits stat analysis if required.

*Details from Instrumentation Laboratory (UK) Ltd, Kelvin Close, Birchwood Science Park, Warrington WA3 7PB, UK. Tel.: 0925 810141.*

**Circle No. 43 on Reader Enquiry Card**

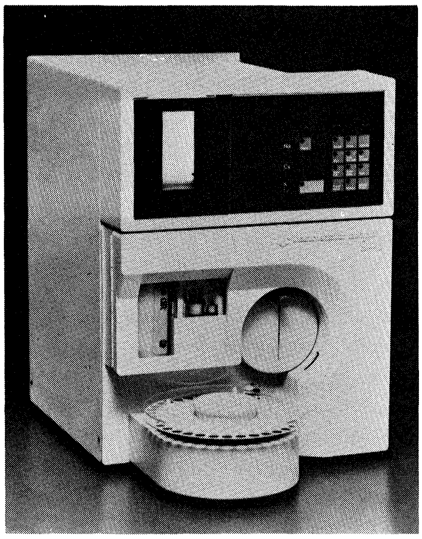

*The IL943: a fully automated, microsampling flame photometer, which has caesium as its internal standard. (Instrumentation Laboratory Ltd, Warrington, UK.)*

### Automatic hypothyroidism screening

The software writers at Innotron Ltd have adapted the company's Hydragamma 16 multiheaded gamma counter to screen automatically for hypothyroidism in newborn infants. The method used is TSH radioimmuno-assay. The Hydragamma has recently been examined using Pharmacia's Phadebas Dry Spot nTSH kit on a range of mock patient samples; the counter was programmed to distinguish patient samples which recorded TSH levels greater than 15 $\mu$ units of nTSH/ml blood. Clinically, such samples may only occur in a very small fraction (less than 1%) of a patient population, but the medical consequences, if left undetected, could be irreversible cretinism.

The Hydragamma's print-out (the machine can achieve a throughput of 200 samples in 7·6 min) marks clearly the screened patient samples which would then be identified: permitting further analysis and medical treatment.

*Full information from Robin Cooper, Innotron Ltd, 2 Avenue Lane, Chapel Street, Cowley Road, Oxford OX4 1EY, UK. Tel.: 0865 726234.*

**Circle No. 44 on Reader Enquiry Card**

### 'Materials Analysis Lab'

Micromeritics have announced a new brochure which describes their Materials Analysis Laboratory's capabilities for particle technology measurements. Sample submission requirements are described and typical analysis results illustrated. Laboratory analyses are available to customers on either a fee or contract basis and consulting services are provided to help customers get the best interpretation of their Micromeritics analyses. Specific analyses offered by the laboratory include particle-size distribution, density and absolute volume, zeta potential, specific surface area, chemisorption, pore structure by automatic physical absorption, mercury intrusion porosity, and mercury contact angle.

*A copy of this brochure can be obtained by writing to Micromeritics Instrument Corporation, 5680 Goshen Springs Road, Norcross, Georgia 30093, USA.*

**Circle No. 45 on Reader Enquiry Card**

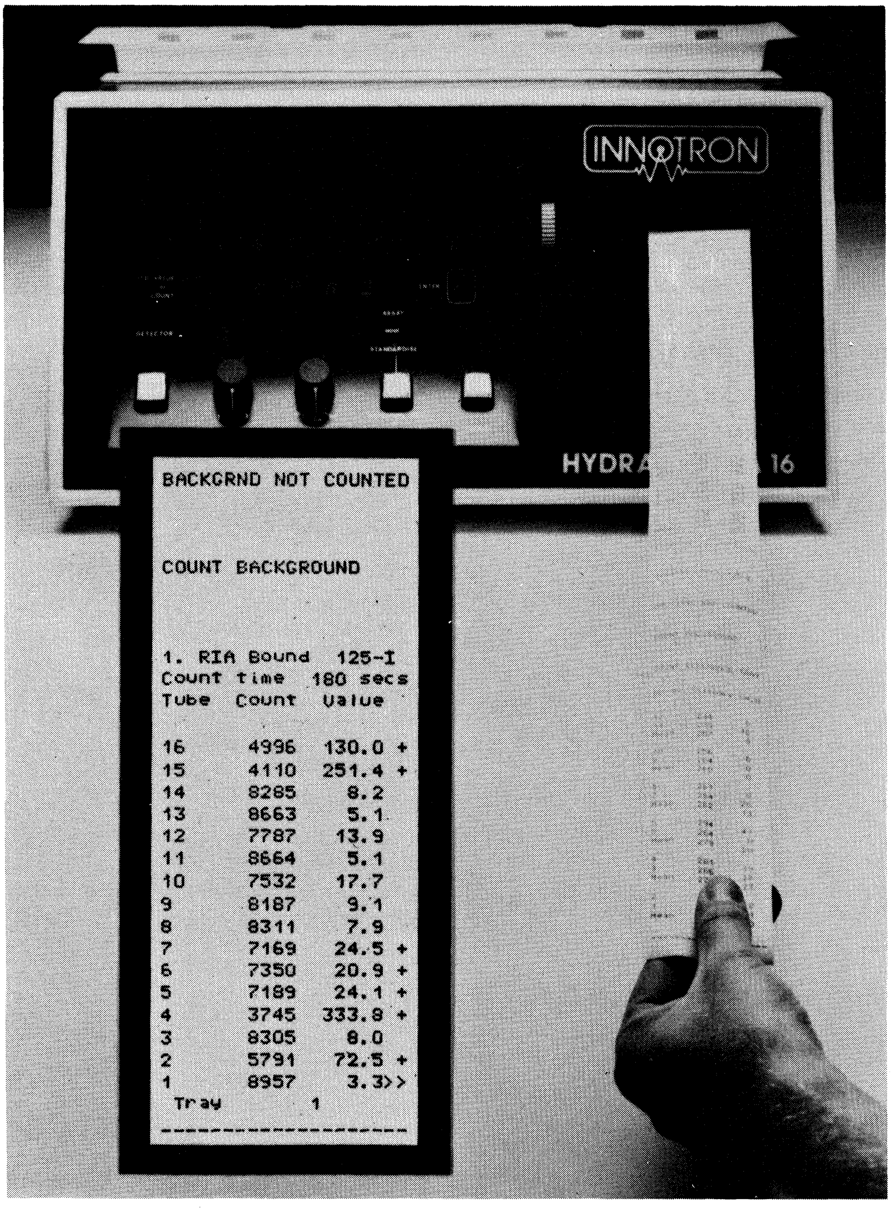

*Innotron's Hydragamma 16—the microprocessor screening facility identifies extraordinary patient samples with a cross.*

### Linomat III for high-precision TLC analysis

The Camag Linomat III, for high-precision narrow band and spot application of samples for quantitative or qualitative TLC analysis, was designed for all TLC users and particularly for those involved in qualitative analysis or preparative work. By using a 'spray-on' technique with nitrogen or compressed air as the carrier gas, large volumes (for example 100 $\mu$l) can be concentrated into a narrow band on the TLC plate. The length of the band can be freely selected between 0 and 199 mm and is evenly distributed. Automatic control of sample dispensing and uniform sample distribution is carried out by separate stepping-motors. With a Hamilton syringe application for a 10$\mu$l sample volume will require between 40 and 100 s (depending on the speed selected and solvent used).

The Linomat III is to be distributed in the UK by Baird & Tatlock Ltd.

*Further information from Baird & Tatlock (London) Ltd, PO Box 1, Romford RM1 1HA, Essex, UK. Tel.: 01 590 7700.*

**Circle No. 46 on Reader Enquiry Card**

### Mass spectrometer at Winter Games

VG Analytical have an order from the International Olympic Committee for an MM7070E mass spectrometer. The machine will be used at the XIVth Winter Games in Sarajevo in 1984 to safeguard against the use of drugs and other non-permitted stimulants by athletes taking part in the competitions. In previous years, less complex quadrupole instruments were quite capable of detecting evidence of drug abuse; however, as drug preparation and application methods have become more and more sophisticated, so the need for extremely accurate, sensitive and reliable monitoring equipment has become more apparent. The MM7070E and a 2350 Data System has clearly been able to satisfy these requirements.

*Information about the MM7070E from VG Analytical Ltd, Tudor Road, Altrincham, Cheshire WA14 5R7, UK. Tel.: 061 928 6300.*

**Circle No. 47 on Reader Enquiry Card**

### Single-well integrity in RIA

The 'Removawells' and 'Removastrips' produced by Dynatech for Microelisa techniques are now being sold for radio immune assay (RIA) procedures. The company has developed a matrix of numbered wellcaps and a simple cap-sealer and press, so researchers are now able to seal a whole plate with pre-numbered caps and then separate each into individual wells. Single-well integrity is thus maintained, which is vital when handling wells during RIA testing. Insertion of the caps is rapid and positive and once the caps have been inserted, the entire plate can be disassembled into individual wells or strips by using the ejection system on the press. Like Dynatech's Microelisa plates, Removawells and Removastrips are constructed from Immulon—a plastic which enhances protein-binding onto the surface of the wells. Removawells consist of 96 individually moulded wells—each identical in format to a single well from a Microelisa plate—held securely in a rigid plastic matrix. Removastrips, specially developed for RIA work, consist of eight wells in strip form, easily separated by either the new press or by a twist of the fingers.

*Further information from Dynatech Laboratories Ltd, Daux Road, Billingshurst, Sussex RH14 9SJ, UK. Tel.: 040381 3381.*

**Circle No. 48 on Reader Enquiry Card**

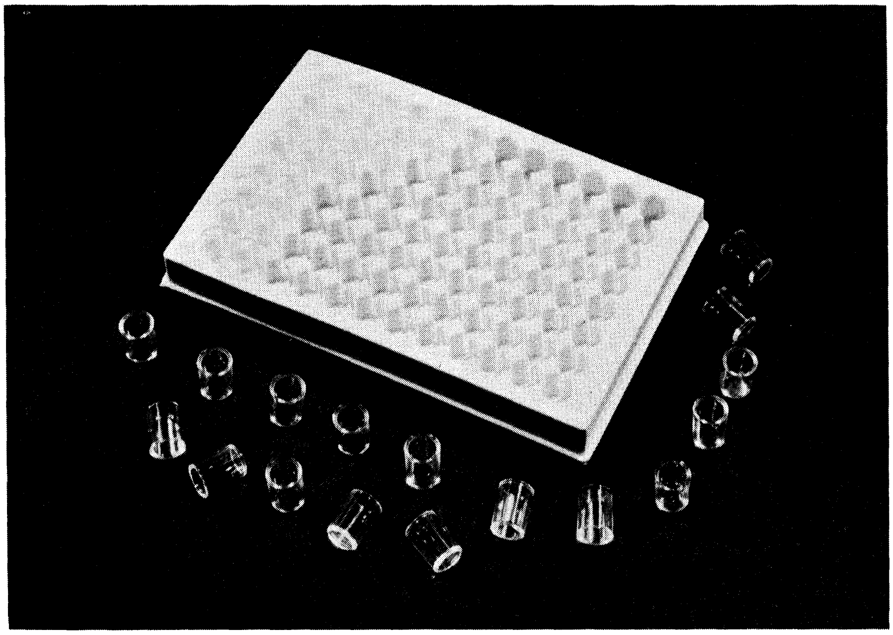

*Dynatech's moulded 'Removawells'—shown both individually and fitted into their rigid plastic matrix.*

### RV100—a viscometer

The Haake RV 100s are the latest generation of rotovisco rotational viscometers. The RV 100 was originally designed for use with the CV 100 low shear viscometer, but its advantages of compactness, convenience and ease of use, mean that it is suitable for anyone who needs a general-purpose rotational viscometer. The RV 100 offers built-in versatile data display and recording and can be operated by almost any laboratory worker.

*Details from MSE Scientific Instruments, Manor Royal, Crawley, Sussex RH10 2QQ, UK. Tel.: 0293 31100.*

**Circle No. 49 on Reader Enquiry Card**

### Epoxy resin quality-control

A new method for the Mettler DL40 memotitrator means that the expoxide value of expoxies can be determined automatically, with complete data analysis and with hard-copy results in any units. The method is loaded and stored in the DL40 and can be recalled at the touch of a key; the DL40 can store up to 20 such methods. High levels of precision can be achieved with the instrument, especially if sample weight data is entered directly from an on-line balance.

*For details of the memotitrator and new method ask MSE Scientific Instruments for 'Mettler Application Bulletin', No. 104.*

**Circle No. 50 on Reader Enquiry Card**

### Automatic titrator

MSE Scientific Instruments have also announced an improvement on the DL40 itself: it now incorporates a 16-place sample changer. Turntable, printer and titrator are co-ordinated by an RT40 microprocessor. Sample weight data is transferred to the titrator from the battery-protected memory which can store up to 50 weights, a balance can be connected on-line for rapid weight-data entry. In addition to titrant, reagent can be added and the burette, stirrer and delivery tips are washed between determinations to prevent carry-over.

The DL40-RT40 combination offers unattended, sequential titration with full data analysis and processing, with a hard-copy record of required sample and method data.

*Details from MSE Scientific Instruments.*

**Circle No. 51 on Reader Enquiry Card**

### The DL40 and Karl Fischer

And MSE are holding a workshop on using the memotitrator with Karl Fischer determinations. Topics to be covered include optimizing the method for different sample and reagent types. There will be ample opportunity for practical work using delegates' own samples. The workshop will be held at MSE's application laboratory in Crawley; dates from MSE.

**Circle No. 52 on Reader Enquiry Card**

### 'Kent Review', Number 9

Two new Kent measurement and control systems are featured in the June 1982 issue of *Kent Review*, Brown Boveri Kent's quarterly house magazine. One of these, a distributed control system in the Kent P4000 range, is capable of controlling up to 4000 plant items with as many as 10 000 individual channels, over distances of up to 5 km. The other—System 19—is a modular instrumentation package for smaller process and analytical measurement and control applications. Other new products discussed in the magazine include a Testomat water-hardness monitor, a non-dispersive infra-red gas analyser in the company's Infragas range, and a microprocessor-controlled multi-stream switching system for EIL ion-selective and colorimetric on-line liquid monitors.

*Copies of 'Kent Review' are available from the Publicity Department, Brown Boveri Kent PLC, Biscot Road, Luton, Bedfordshire LU3 1AL, UK. Tel.: 0582 21151.*

**Circle No. 53 on Reader Enquiry Card**

### The first stand-alone mass-selective detector for chromatographers

A team from Hewlett-Packard's Analytical Group introduced a new, stand-alone gas chromatographic detector for specific and selective sample-component identification at Laboratory '82, which was held in London in mid September. The machine has been designated the HP 5970A mass-selective detector (MSD). The machine can be interfaced with most capillary gas chromatographs and is intended for the gas-chromatograph user who needs fast, low-cost sample identification. Used specifically in capillary gas chromatography, the HP 5970A makes chemical analyses in such areas as methods development for confirmation of target-compound identity, drug contents and stat identification in overdose cases. It will also be important in pesticide analyses, providing data specifically comparable to, or better than, that obtained with nitrogen-phosphorus, flame-photometric and electron-capture detectors.

The HP 5970A provides both qualitative and quantitative information about a sample. It confirms the presence of an analyte through spectral acquisition when used in qualitative analyses. And, due to selected-ion-monitoring (SIM) software, it monitors up to six ions with a high degree of specificity. Simultaneous quantitation of selected ions may be performed, and, with due consideration to the inherent limitations of quadrupole-based analysers, the system's quantitative performance approaches that of conventional detector/integrator systems.

The HP 5970A MSD system is supplied in two compact modules. Attachable to either the right or the left side of the gas chromatograph, the detector module houses the hyperbolic quadrupole mass analyser, an air-cooled turbo-molecular pump and other related hardware. In addition, the module is easy to maintain and requires no special utilities for operation. The second controller module includes an HP 9825B computer controller mounted with an HP 2671G graphics printer.

Software for spectral-data acquisition, SIM and internal diagnostics are standard with the machine, as is software which allows users to design and input custom programs. Specifications for the HP 5970A MSD unit include operation on 120 or 240V, a mass range of 10–600 amu, five-decade dynamic range and capillary-direct capability to allow sample components to elute directly into the detector-ion source. The detector module measures 39·0 x 64·0 x 64·0 cm. Options include open-split interfaces, which allow narrow- or wide-bore, fused-silica capillary columns of any type to be used.

*Details from Enquiries Section, Hewlett-Packard Ltd, Winnersh, Wokingam, Berkshire RG11 5AR, UK. Tel.: 0734 784774.*

**Circle No. 54 on Reader Enquiry Card**

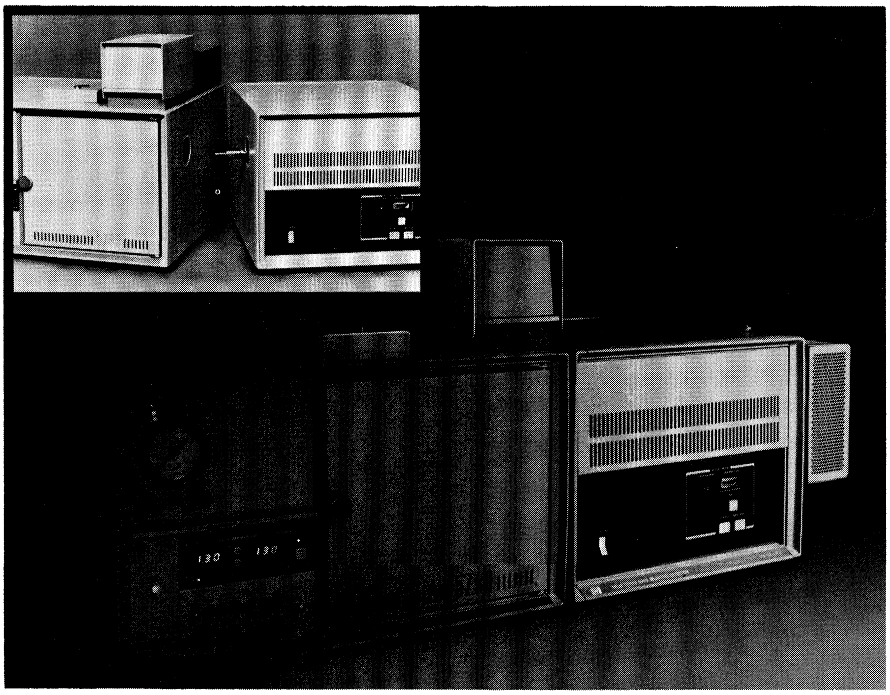

*The HP 5970A, a low-price mass-selective detector, which offers chromatographers high sensitivity, selectivity and unambiguous compound identification. The inset shows how the detector interfaces to the capillary column through an opening in the gas chromatograph oven. (Hewlett-Packard Ltd, Wokingham, UK.)*

### Blood-cell counter

Contraves Instruments' 16-parameter haematology analyser (the Contraves 8016) is to be sold in the UK by Baird & Tatlock. Most multi-parameter instruments cost over £40 000 but this one is priced at under £19 000 putting it within reach of many laboratories. Allied to the new Contraves autodilutor 8010, a throughput of 60 samples/h can be achieved, so it is ideally suited for back-up, on-call, and medium-size work-loads of 100 samples/day. '8016' means eight or 16 parameters. The standard eight-parameter print-out is for WBC, RBC, Hb, HCT, MCT, MCH, MCHC, and platelets, and by keyboard command RDW, RDS, MPV, PCT, PDW, PDS, LYC and LYM can be analysed and histograms of WBC, RBC and platelets generated.

The instrument features threshold settings, automatic detection of system error, print-out of instruction for error-tracking, automatic capillary black-flush, a built-in service programme and automatic evaporation of animal blood or other particle suspensions. An EDp interface is available.

*More information from Mr C. Burton, Baird & Tatlock (London) Ltd, PO Box 1, Romford RM1 1HA, Essex, UK. Tel.: 01 590 7700.*

**Circle No. 55 on Reader Enquiry Card**

### Digital display

The Digi-King series, newly launched by Pope Scientific Inc., is a range of giant-size digital display equipment. The numbers on the units are $3\frac{1}{4}$ in high and are legible from 100 ft; signals from transducers, voltage or current sources, contact closures or solid-state devices

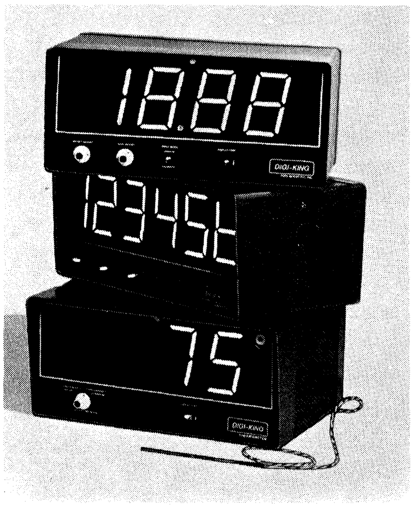

*Digi-Kings—display units with $3\frac{1}{4}$ in high digits, in red or in green. Cabinet dimensions of Digi-Kings are $14\frac{5}{8}$ in (width) × 7 in (height) × $10\frac{1}{4}$ in (depth); they weigh between 14 and 17 lb (depending on the model); power requirements are 110 V a.c., 50/60 Hz, at 1 A.*

are automatically converted to desired measurements. There are six Digi-Kings on the market:

(1) An analogue-to-digital converter, which displays most linear parameters that can be expressed in −1999 to +1999 units; the signal is automatically converted.

(2) A BCD signal indicator, which accepts BCD signals from other instruments and displays corresponding numbers; coded card edge connectors are used.

(3) A counter/timer, which counts up any series of events that can provide an appropriate signal. Elapsed time is displayed in seconds or tenths of a second and 'hold' and 'reset' functions are included. Readings up to 2 000 000 are possible.

(4) A programmable up-down counter/timer—this has all the features of the counter/timer, plus a selector switch for up or down count; a pre-settable starting point, so that count can be started and reset at any desired number; and an output signal for use as a controller when the count reaches zero.

(5) A rate indicator and counter, which can display rate of virtually any series of operations that can provide a contact closure or electronic signal for each event. This Digi-King automatically updates the rate at the end of the time increment. A switch provides a cumulative total and 2 000 000+ are possible.

(6) A thermometer which covers the range of −55°C to +150°C; centigrade, Fahrenheit, Kelvin or Rankine scales can be displayed.

*More information from Whitney Nichols, Pope Scientific Inc., PO Box 495, Menomonee Falls, Wisconsin 53051, USA. Tel.: 414 251 9300.*

**Circle No. 56 on Reader Enquiry Card**

### GC/LC and GPC chromatography data systems

Anaspec now offer a range of chromatography data systems. Systems are available for GC/LC and GPC applications with software facilities allowing real-time capture and display of chromatograms, calculation of results (peak heights, retention times, percentage area, base-line correction) and the ability to store the raw data on cassette or disc.

All of the systems are based on Hewlett-Packard's range of microcomputers and allow greater flexibility than purpose-built integrators. A new feature is multi-instrument capability, up to 10 chromatographs can be simultaneously controlled by one computer system, with further facilities to control timed events and autosampling standard on all systems. The software packages are menu-driven with prompts aimed at the chromatographer rather than a computer expert, so there is no need for the user to understand a programming language.

*Details from Anaspec Ltd, Pearl House, Bartholomew Street, Newbury, Berkshire RG14 5LL, UK. Tel.: 0635 44329.*

**Circle No. 57 on Reader Enquiry Card**

### 'Liquid Scintillation Supplies Handbook and Selection Guide'

Having fairly recently introduced several new instruments for liquid scintillation counting, Beckmann-RIIC have published a 34-page brochure (their Bulletin 7397) called *Liquid Scintillation Supplies Handbook and Selection Guide.*

The booklet will be of interest to anyone involved in liquid scintillation counting: it contains details of how to select the correct 'cocktail' for use, as well as a guide to the selection of the most suitable vials. Beckman cocktails and vials are also listed in some detail, together with ingredients. Special applications are discussed in the brochure, which is illustrated with useful graphs of counting efficiences and phase diagrams for a wide variety of sample types for each cocktail.

*This brochure is available free of charge from Beckman-RIIC Ltd, Sands Industrial Estate, Progress Road, High Wycombe, Buckinghamshire, UK. Tel.: 0494 41181.*

**Circle No. 58 on Reader Enquiry Card**

### 'CAMAG Bibliography Service'

CAMAG produce a biannual bulletin, called *CAMAG Bibliography Service*, which abstracts a number of scientific and technical papers on thin-layer chromatography. The editor, Dieter Jänchen, has split the abstracts into TLC books; review articles; papers on theory, instruments, separation media, methods, and into the analysis of particular chemistries. Issue 49, which was sent to *JAC* for mention, has about 200 entires with summaries averaging around 50 words. The *Bibliography Service* covers the literature admirably—there are abstracts here from the journals one would expect: *Journal of Liquid Chromatography, Analytical Chemistry, Journal of Chromatography, Chromatographia, Journal of High Resolution Chromatography* etc., but the not so easily acquired East European and specialist publications are also scanned. The *Service* should interest all TLC workers.

*Details from Ch. Gfeller, CAMAG, Sonnenmattstrasse 11, CH-4132 Muttenz, Switzerland.*

**Circle No. 59 on Reader Enquiry Card**

